# Ursolic acid induces cell cycle arrest and apoptosis of gallbladder carcinoma cells

**DOI:** 10.1186/s12935-014-0096-6

**Published:** 2014-10-29

**Authors:** Hao Weng, Zhu-Jun Tan, Yun-Ping Hu, Yi-Jun Shu, Run-Fa Bao, Lin Jiang, Xiang-Song Wu, Mao-Lan Li, Qian Ding, Xu-an Wang, Shan-shan Xiang, Huai-Feng Li, Yang Cao, Feng Tao, Ying-Bin Liu

**Affiliations:** Department of General Surgery, Xinhua Hospital, Affiliated to Shanghai Jiao Tong University, School of Medicine, Shanghai, China; Laboratory of General Surgery, Xinhua Hospital, Affiliated to Shanghai Jiao Tong University, School of Medicine, Shanghai, China; Institute of Biliary Tract Disease, Shanghai Jiao Tong University School of Medicine, No. 1665 Kongjiang Road, Shanghai, 200092 China; Gastrointestinal Surgery, Shaoxing People’s Hospital Shaoxing Hospital of Zhejiang University, No. 568 Zhongxing North Road, Shaoxing, 312000 Zhejiang Province China

**Keywords:** Ursolic acid, Gallbladder cancer, Proliferation, Cell cycle, Apoptosis, Mitochondrial-mediated pathway

## Abstract

**Background:**

Ursolic acid (UA), a plant extract used in traditional Chinese medicine, exhibits potential anticancer effects in various human cancer cell lines *in vitro*. In the present study, we evaluated the anti-tumoral properties of UA against gallbladder carcinoma and investigated the potential mechanisms responsible for its effects on proliferation, cell cycle arrest and apoptosis *in vitro*.

**Methods:**

The anti-tumor activity of UA against GBC-SD and SGC-996 cells was assessed using MTT and colony formation assays. An annexin V/PI double-staining assay was used to detect cell apoptosis. Cell cycle changes were detected using flow cytometry. Rhodamine 123 staining was used to assess the mitochondrial membrane potential (ΔΨm) and validate UA’s ability to induce apoptosis in both cell lines. The effectiveness of UA in gallbladder cancer was further verified in vivo by establishing a xenograft GBC model in nude mice. Finally, the expression levels of cell cycle- and apoptosis-related proteins were analyzed by western blotting.

**Results:**

Our results suggest that UA can significantly inhibit the growth of gallbladder cancer cells. MTT and colony formation assays indicated dose-dependent decreases in cell proliferation. S-phase arrest was observed in both cell lines after treatment with UA. Annexin V/PI staining suggested that UA induced both early and late phases of apoptosis. UA also decreased ΔΨm and altered the expression of molecules regulating the cell cycle and apoptosis. In vivo study showed intraperitoneally injection of UA can significantly inhibited the growth of xenograft tumor in nude mice and the inhibition efficiency is dose related. Activation of caspase-3,-9 and PARP indicated that mitochondrial pathways may be involved in UA-induced apoptosis.

**Conclusions:**

Taken together, these results suggest that UA exhibits significant anti-tumor effects by suppressing cell proliferation, promoting apoptosis and inducing 7cell cycle arrest both *in vitro* and *in vivo*. It may be a potential agent for treating gallbladder cancer.

## Background

**Figure 1:**
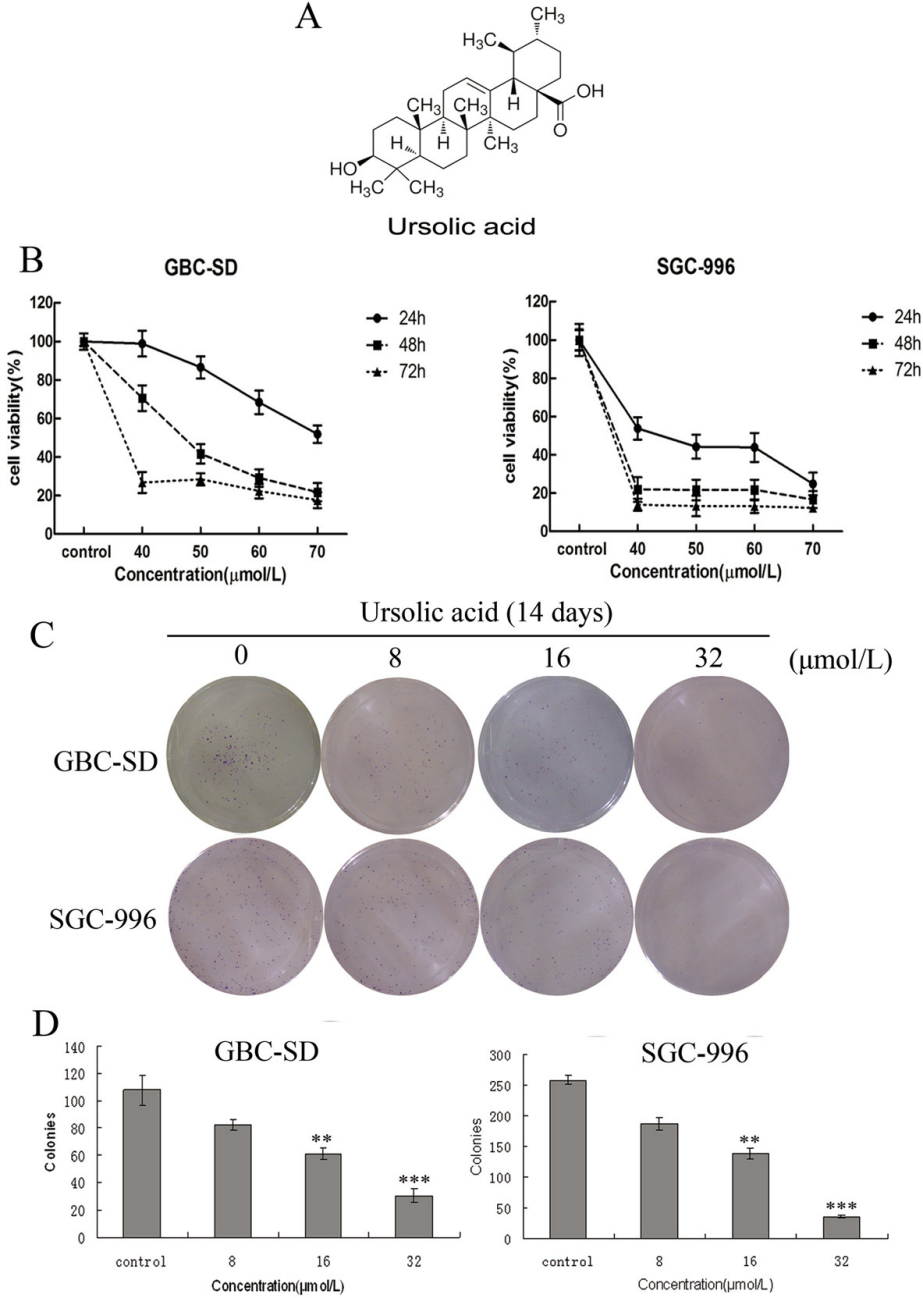
**UA inhibits proliferation in GBC cells. (A)** The chemical structure of UA. **(B)** GBC-SD and SGC-996 cells were treated with various concentrations of UA for 24, 48 or 72 h. Effects on cell proliferation were determined using a MTT assay. Each value represents the mean ± SD (n = 3). **(C-D)** UA inhibits colony formation in GBC cells. GBC-SD and SGC-996 cells were treated with different doses of UA (8, 16 or 32 μmol/L) and were allowed to form colonies in fresh medium for 14 days. The photomicrographic differences and number of colonies (mean ± SD, n = 3) in colony formation are shown. Significant differences from the control are indicated by *p < 0.05 and **p < 0.01.

Gallbladder cancer (GBC) is the most common malignancy of the biliary tract and the 5th most common digestive tract cancer [1]. Owing to a lack of specific signs and symptoms, many patients are not diagnosed until the cancer has reached an advanced stage, with local invasion and distant metastasis. As a result, GBC patients have poor prognoses, and the 5-year survival rate is approximately 5% [2]. Surgical resection is the only potentially effective treatment for GBC, but recurrence rates remain high, even after radical resection [3]. In addition, GBC is resistant to chemotherapy or radiotherapy, hindering the prevention of tumor development and recurrence. Novel and more effective chemotherapeutic agents are urgently needed to treat GBC. Ursolic acid (UA) (Figure 1A) is a pentacyclic triterpene acid that can be extracted from a number of plants, including fruits and medicinal herbs, and has a variety of clinical applications. In addition to its antioxidant, anti-hepatitis, anti-inflammatory and hypolipidemic effects, the most remarkable property of UA is its anti-tumor activity. UA inhibits cell growth and induces apoptosis in several cancers, including colon cancer, breast cancer, leukemia, melanoma and prostate cancer [4-8]. This cell growth inhibition is mediated by BAX through the suppression of the PI3K/Akt pathway. UA downregulates the expression of STAT3-regulated gene products such as cyclin D1, Bcl-2, Bcl-xL, survivin, Mcl-1 and VEGF in multiple myeloma cells [9]. Other studies have suggested that UA induces apoptosis through caspase-3 activation concomitant with a significant decrease in Bcl-2 and survivin expression [10]. UA also inhibits JNK expression and IL-2 activation in Jurkat leukemic T cells, resulting in reduced proliferation and T cell activation [11]. Despite considerable research on the effects of UA on various cancers, no detailed studies have investigated the effects of UA on human gallbladder carcinoma. In the present study, we confirm the anti-neoplastic activity of UA in GBC cell lines (including GBC-SD and SGC-996) and provide insight into the molecular mechanisms underlying this activity. Its tumor growth inhibition effectiveness was further verified by in vivo study.

## Results

### Effects of UA on the viability of gallbladder carcinoma cell lines

According to the MTT assay results shown in Figure 1B, the proliferation of both GBC-SD and SGC-996 cells was inhibited after treatment with different concentrations of UA for different periods. Furthermore, inhibition was dose- and time-dependent. The groups at 48 h were chosen to detect changes in molecular events during the subsequent experiments. The IC50 values in GBC-SD and SGC-996 cells at 48 h were approximately 47.6 μmol/L and 28.5 μmol/L, respectively.

### Effects of UA on colony formation by gallbladder carcinoma cell lines

The ability of GBC-SD and SGC-996 cells to form colonies in the presence of UA was analyzed using a flat plate colony formation assay. The results shown in Figure 1C suggest that UA effectively inhibited colony formation in both cell lines in a dose-dependent manner. Moreover, statistical analysis demonstrated that the mean sizes of the control colonies were larger than the colonies of the drug-treated groups (Figure 1D). This finding suggests that UA may significantly affect GBC-SD and SGC-996 cell proliferation.

### UA-induced cell cycle arrest and apoptosis in gallbladder carcinoma cell lines

**Figure 2:**
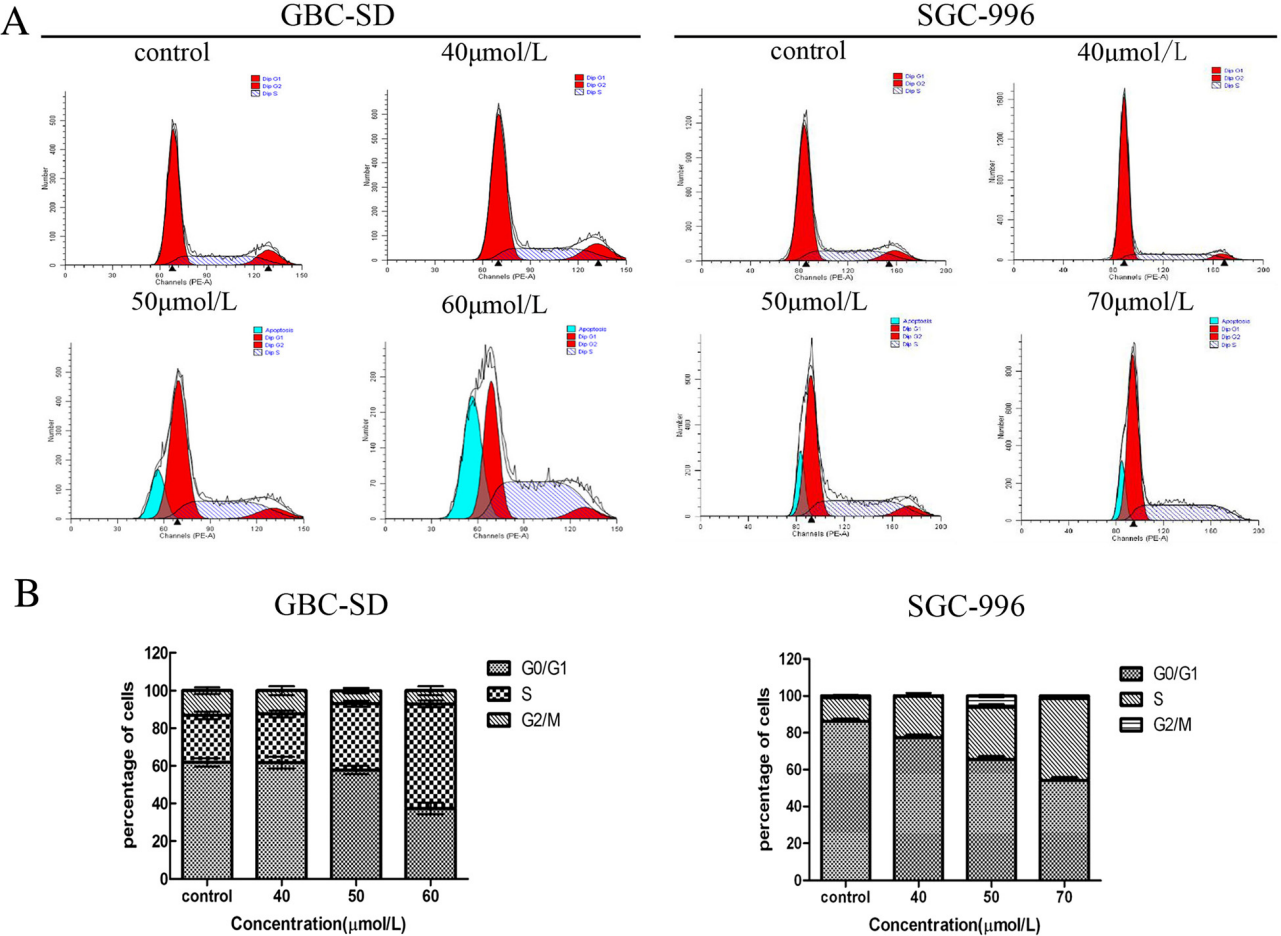
**UA induces cell cycle arrest at the S phase in GBC cells.** GBC-SD and SGC-996 cells were treated with different concentrations of UA for 48 h. **(A)** The cell cycle distribution of treated cells was determined using flow cytometry. **(B)** The data are expressed as the mean ± SD (n = 3), with results representative of 3 independent experiments shown. *p < 0.05, **p < 0.01 and ***p < 0.001 vs. the control group.

Flow cytometry was used to determine the effects of UA on cell cycle distribution and apoptosis; the results are shown in Figure 2. After treatment with UA for 48 h, the percentage of S-phase cells in GBC-SD colonies was significantly higher than in the control group (36.84% and 51.12% in the 50 μmol/L and 60 μmol/L groups, respectively, versus 26.72% in the control group). In addition, the percentage of S-phase cells in the SGC-996 cell line was 16.38% in the control group, 21.40% in the 40 μmol/L group, 30.52% in the 50 μmol/L group and 41.63% in the 70 μmol/L group. Both cell lines achieved peak apoptosis once they were treated with > 50 μmol/L UA. These results suggest that UA arrests the cell cycle at the S phase and induces apoptosis *in vitro*.

### Flow cytometric estimation of UA-induced apoptosis

**Figure 3:**
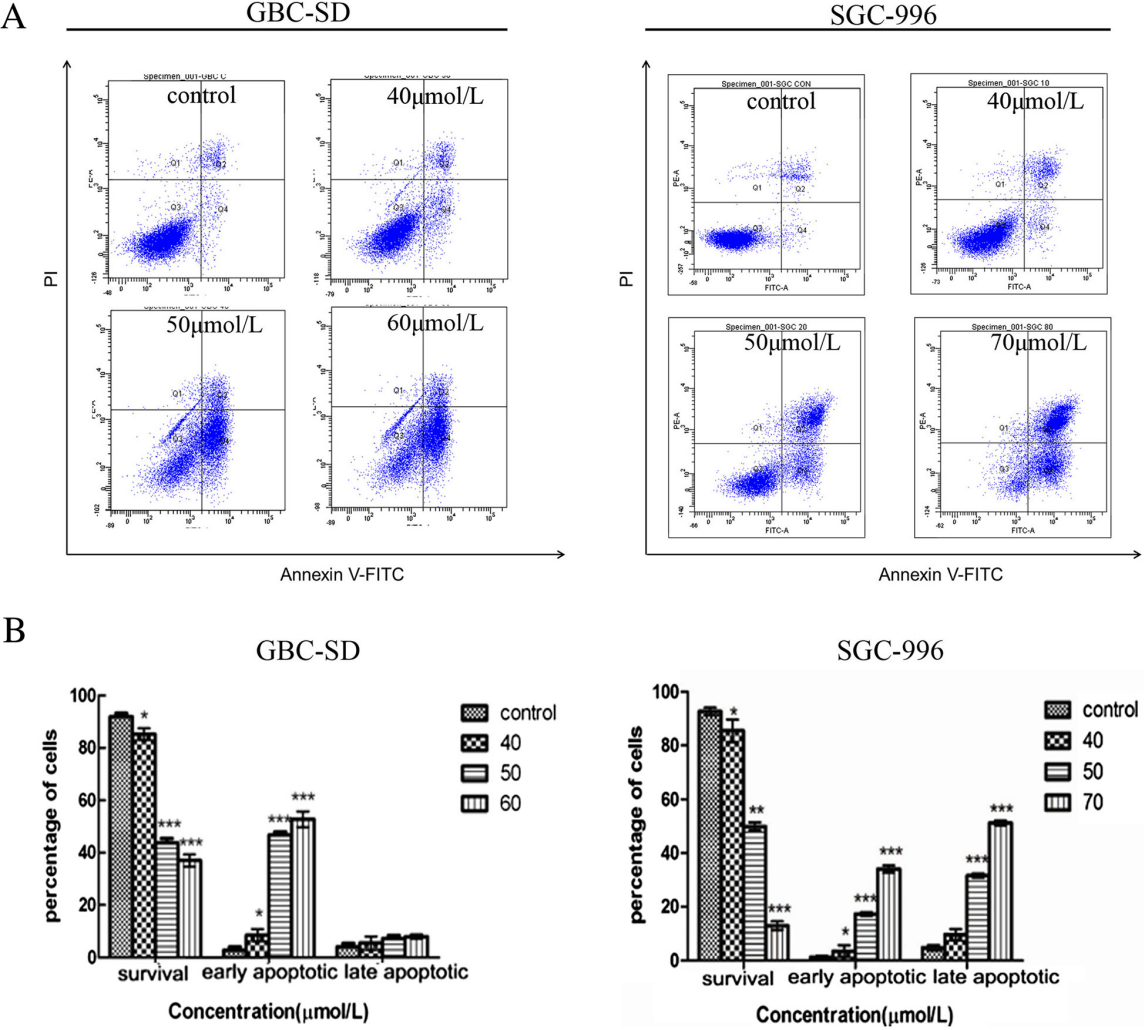
**UA induces apoptosis in GBC cells.** GBC-SD and SGC-996 cells were treated with different concentrations of UA for 48 h. **(A)** Flow cytometric analysis of UA-induced apoptosis in GBC cells using annexin V-FITC/PI staining. Cells in the lower right quadrant represent early apoptotic cells, and those in the upper right quadrant represent late apoptotic cells. **(B)** The percentage of apoptotic cells is presented as the mean ± SD. The data are representative of 3 similar experiments. Significant differences from the control are indicated by *p < 0.05, **p < 0.01 and ***p < 0.001.

Phosphatidylserine (PS) is located inside normal cells and is transferred to the surface during the early stage of cell apoptosis. Annexin V, a Ca^2+^-dependent phospholipid binding protein, has a strong binding affinity for PS. Thus, we used an annexin V-FITC/PI staining kit to assess UA-induced cell apoptosis. The results shown in Figure 3 indicate a remarkable dose-dependent increase in both the early and late stages of apoptosis in both cell lines compared with the control group.

### Effects of UA on ΔΨm

**Figure 4:**
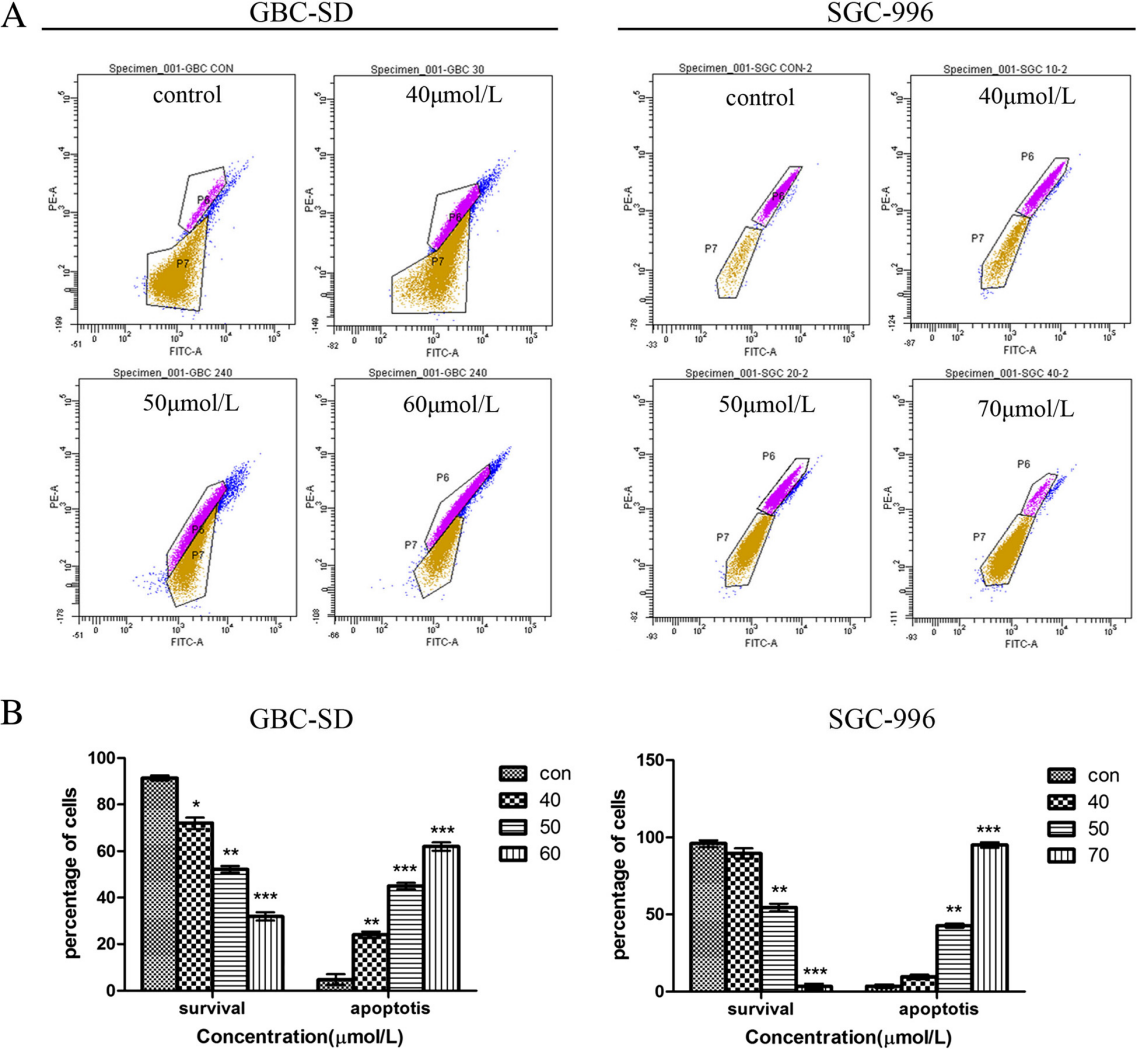
**UA affects ΔΨ** **m in GBC cells.** GBC-SD and SGC-996 cells were treated with UA for 48 h and then stained with the membrane-sensitive probe Rhodamine 123. **(A)** Rhodamine retention was measured by flow cytometry. The results are representative of 3 independent experiments. **(B)** The corresponding linear diagram shows the percentages of Rhodamine 123-negative cells as the mean ± SD (n = 3). *p < 0.05, **p < 0.01 and ***p < 0.001 vs. the control group.

To validate the ability of UA to induce apoptosis in GBC-SD and SGC-996 cells, we performed a cellular functional assay. Rhodamine 123 staining was used to detect the integrity of the mitochondrial membrane. Loss of ΔΨm was correlated with a decrease in the intensity of fluorescent staining. As shown in Figure 4, after UA treatment, the ratio of Rhodamine 123-negative cells increased from 3.56% to 63.3% in GBC-SD cells and from 5.63% to 98.8% in SGC-996 cells. This increase occurred in a dose-dependent manner, suggesting that UA induces cancer cell apoptosis through a mitochondria-dependent mechanism.

### Effects of UA on the signal pathway of caspase and Bcl-2 family members

**Figure 5:**
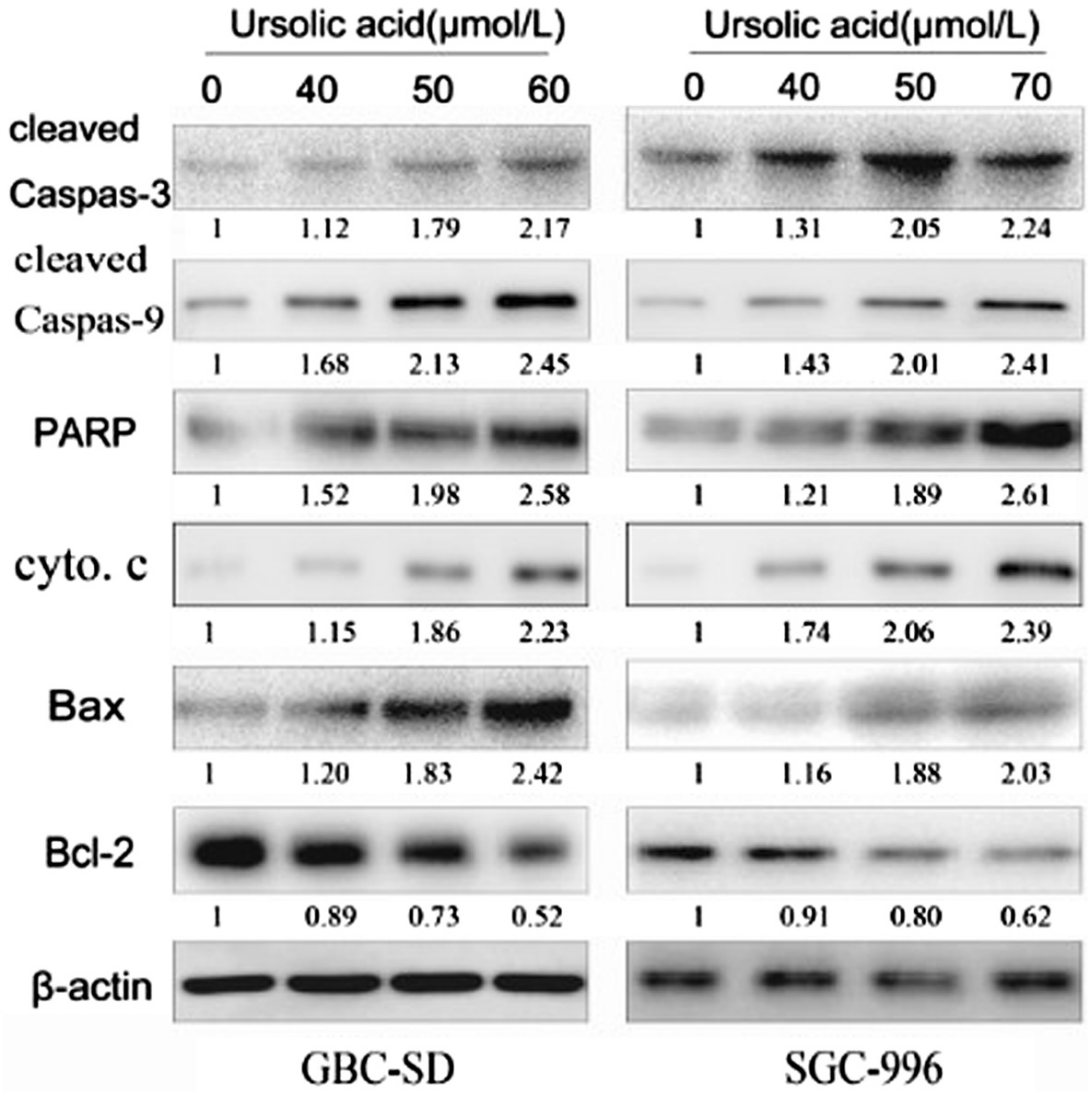
**UA modulates the expression of cell cycle- and apoptosis-related proteins in GBC cells.** Western blot analysis of protein extracts from GBC-SD and SGC-996 cells treated with different doses of UA for 48 h. The expression levels of cleaved caspase-3, caspase-9, PARP, cyto c, Bax and Bcl-2 were analyzed. β-Actin was used as a loading control. The results are representative of 3 independent experiments.

To investigate the molecular mechanism underlying the apoptotic effects of UA on gallbladder carcinoma cells, we assessed the expression of several apoptosis-related proteins, including PARP, caspase-3, caspase-9, cytochrome c, Bax and Bcl-2, by western blot analysis. As shown in Figure 5, as the concentration of UA increased, Bax protein expression increased, while Bcl-2 expression decreased. The Bax/Bcl-2 ratio increased in both cell lines, suggesting that these proteins are involved in UA-induced apoptosis. In the caspase pathway, cleaved caspase-3, −9 and PARP levels were upregulated after treatment with UA in a dose-dependent manner. Caspase-3 is a major functional enzyme in the cellular apoptotic signaling pathway, which suggests that UA may induce GBC cell apoptosis via caspase-3 activation.

### UA suppresses tumor growth in vivo

**Figure 6:**
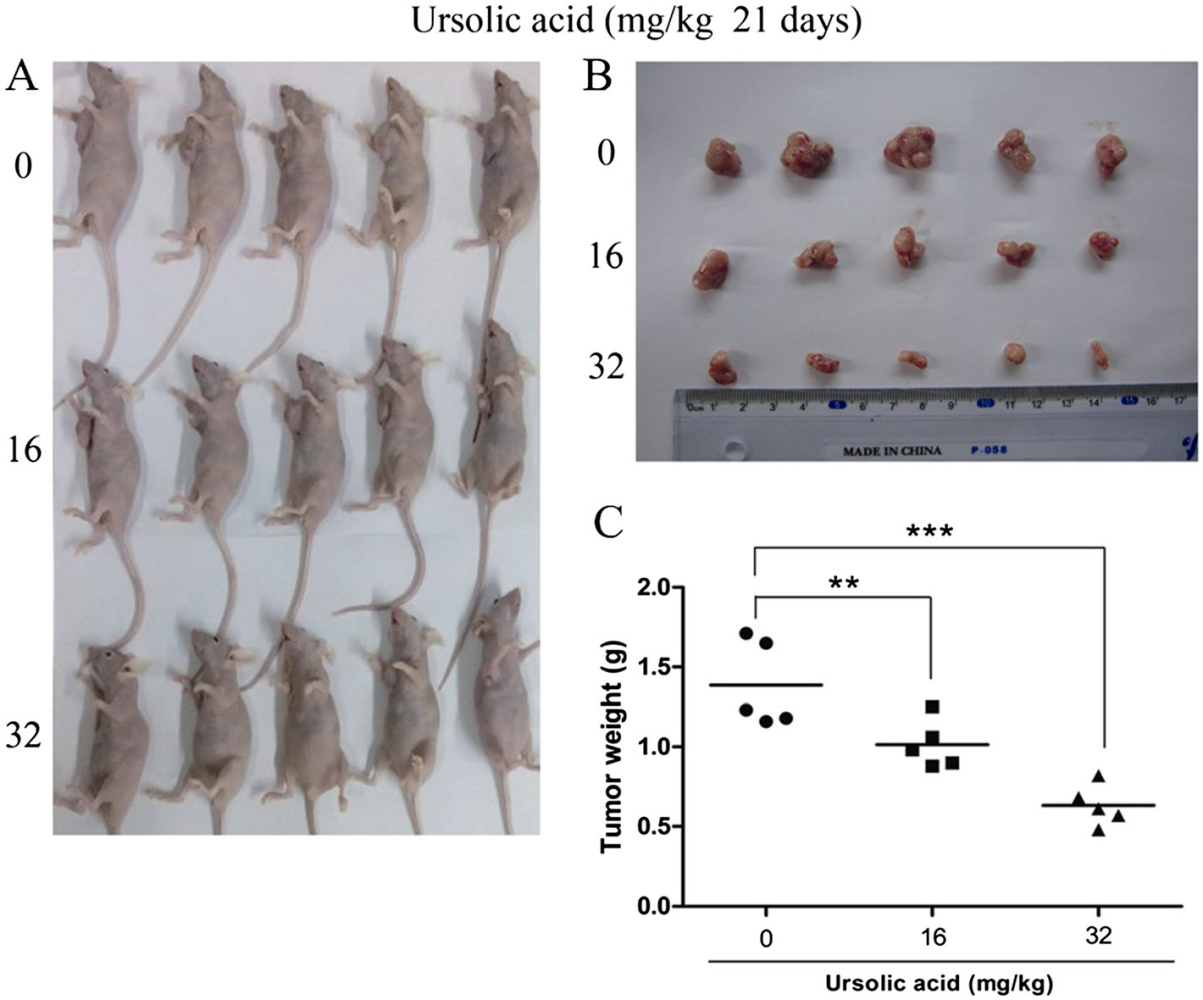
**UA suppressed the growth of tumor in nude mice injected with GBC-SD cells. (A)** GBC-SD cells were subcutaneously injected into the right flank of the nude mice; The mice were then administered 0.2 mL of vehicle (10% DMSO and 90% PBS) or UA (16 mg/kg and 32 mg/kg) intraperitoneally everyday for up to 22 days. Photos of 5 representative mice (n = 10) from each group were presented to show the sizes of the resulting tumors; **(B,C)** Tumors were excised from the animals and weighed. *P < 0.05 **P < 0.01 vs. the control group.

We successfully established GBC xenograft model in 45 nude mice. To evaluate the anti-tumor effect of UA, mice in 3 groups were administered UA or vehicle solvent for 21 days.The tumors removed from these mice are shown in Figure 6A, B, and their mean weights were provided in Figure 6C. Compare to the control group, the tumor volume and weight in UA treated group was significantly reduced, and the reduction is dose related. These results accord with our *in vitro* study and further confirmed the effectiveness of UA in gallbladder cancer.

## Discussion

Most malignant tumors remain incurable and are associated with a poor prognosis. Traditional Chinese medicine (TCM) treats cancer in a unique way compared to Western medicine. For example, in TCM, the body as a whole is emphasized, and treatment is focused on modulating the body’s internal environment, including “Blood Qi” flow and “Yin-Yang” balance [12]. These concepts do not exist in Western medicine, and thus, therapeutic effects related to these ideas are not widely accepted.

Recently, the molecular anti-tumor mechanisms of a large catalogue of traditional Chinese drugs were investigated, revealing that these drugs have the same anti-tumor properties as drugs used in Western medicine, expanding our understanding of TCM and chemotherapy [13-16].

The process of tumor development requires multiple steps, including cell initiation, proliferation, invasion and metastasis [17,18]. We have previously identified several other medicines that inhibit tumor cell proliferation and induce apoptosis, thereby influencing the process of tumor development [17,19]. In the present study, we investigated the anti-tumoral properties of UA. The results of cytology and proteomics experiments allow us to conclude for the first time that UA has anticancer properties in GBC cells similar to those previously observed for other cancer cell types.

The drug’s cytotoxicity was evaluated using MTT and colony formation assays. The MTT assay results indicated that at concentrations > 40 μmol/L, UA significantly inhibited GBC-SD and SGC-996 cell growth in a time- and dose-dependent manner. The SGC-996 cells were more sensitive, and an exposure time of 48 h was determined to be the most suitable for subsequent experiments. In the colony formation assays, a smaller dose of UA (>16 μmol/L) effectively inhibited colony formation in both cell lines. Taken together, these results indicate that UA suppresses cancer cell growth. To better understand the effect of UA, flow cytometric analysis was performed. The results of this analysis suggested that UA causes S-phase arrest in a dose-dependent manner. Cell cycle arrest may be the mechanism by which UA inhibits the proliferation of cancer cells.

Apoptosis is an area of intense interest in cancer research. The process of programmed cell death involves a cascade of molecular events that are initiated by several stimuli [20]. After confirming the apoptosis-inducing effects of UA by flow cytometry, we examined the variation in ΔΨm, as mitochondria play an important role in regulating many cellular functions. During the early stage of cell apoptosis, the permeability of the mitochondrial membrane is increased, consequently decreasing ΔΨm. Our study suggests that UA-induced apoptosis is closely related to this decrease in ΔΨm.

The mitochondrial pathway is one of the three major pathways involved in apoptosis, and NF-κB is a critical transcription factor that regulates the transcription of many genes associated with tumorigenesis [21]. Its target gene, Bcl-2, is also a central regulator of this process. Bcl-2 family proteins play key roles in controlling the mitochondrial pathway [22,23]. The Bcl-2 family is divided mainly into Bax, Bcl-2 and Bid proteins based on their different biological effects. Bcl-2 is regarded as a key apoptosis inhibitor which binds to the mitochondrion and prevent the release of cytochrome c from the mitochondria. On the other hand, Bax acts as an apoptosis promoter via increase the permeability of the mitochondria, which leads to membrane potential loss and cytochrome c releasing. The destiny of a cell is determined by the ratio of these two proteins, Bcl-2/Bax. In the present study, the Bcl-2/Bax ratio was decreased by treatment with UA, causing the elevation level of cytochrome c in the cytosol. Which suggests that UA suppresses NF-κB nuclear localization and changes the proportion of pro-apoptotic and anti-apoptosis proteins in Bcl-s family to induce tumor cell apoptosis.

As the Bcl-2/Bax ratio decreases, it can also cause caspase activation and PARP cleavage.Caspase-9 is activated in the mitochondria-mediated intrinsic pathway. It can subsequently activate Caspase-3. Caspase-3 is known as the “executor of apoptosis”. It can mediates apoptosis in many human cells and in many ways, such as by degrading anti-apoptosis proteins and cleaving DNA repair molecules, extracellular matrix proteins, skeleton proteins and other related molecules [24]. Once activated, caspase-3 can systematically dismantle cells by cleaving key proteins such as PARP. The changes in cleaved caspase-3 and −9 expression observed in our study were consistent with the changes in cell apoptosis observed after treatment with UA. PARP cleavage increased accordingly, suggesting the involvement of a caspase-dependent pathway via caspase-3 in UA-induced apoptosis.

## Conclusion

In summary, our results indicate that UA exhibits potent anti-GBC properties by suppressing cell proliferation, promoting apoptosis and inducing cell cycle arrest *in vitro*. It can also suppresses tumor growth *in vivo*. Activation of the mitochondrial-mediated apoptosis pathway is one possible mechanism responsible for these effects. Our work provides a new perspective on the function of UA in tumor chemotherapy, and we believe that further exploration of similar TCM drugs will reveal additional chemotherapeutic mechanisms.

## Methods

### Drugs and antibodies

UA was purchased from Sigma-Aldrich (St Louis, MO, USA) in the purity of 99.56% and dissolved in dimethyl sulfoxide (DMSO). The stock solution was stored at −20°C. Before use, the drug was defrosted and diluted with cell culture medium to different concentrations. The following antibodies were used: rabbit reactive monoclonal anti-cleaved caspase-3, anti-PARP, anti-Bcl-2 and anti-Bax antibodies and a mouse anti-β-actin antibody. All antibodies were obtained from Cell Signaling Technology (Danvers, MA, USA).

### Cell lines and culture

The human cell lines GBC-SD and SGC-996 were purchased from the Shanghai Cell Institute Country Cell Bank. GBC-SD cells were cultured in high-glucose DMEM (Gibco, USA) at 37°C in a 5% CO_2_ incubator, while SGC-996 cells were cultured in Roswell Park Memorial Institute (RPMI) 1640 medium. Both types of culture medium were supplemented with 10% fetal bovine serum (Gibco, USA), penicillin (100 U/mL) and streptomycin (100 U/mL) (Utah, HyClone, USA).

### UA cytotoxicity assay

A drug cytotoxicity assay was performed using the MTT method. In brief, GBC-SD and SGC-996 cells were placed in 96-well culture plates at a density of 5 × 10^3^ cells per well. After culturing overnight, the initial culture medium was removed, and fresh medium containing various concentrations of UA (0, 40, 50, 60 or 70 μmol/L) were added. The cells were further cultured for 24, 48 or 72 h. Then, 40 μL of 5 mg/mL MTT in PBS was added to each well and incubated for another 4 h. Next, 100 mL of DMSO was used to dissolve the formazan crystals that formed. Cell proliferation was evaluated by measuring the optical density (OD) at 490 nm using an Automated Microplate Reader (Bio-Tek, USA). The percentage of viable cells was calculated using the following formula: cell viability (%) = (OD of treated cells/OD of control cells) × 100. All experiments were repeated three times.

### Colony formation assay

GBC-SD and SGC-996 cells in the logarithmic growth phase were digested using trypsin-EDTA (Gibco, USA) solution and then seeded in 6-well culture plates at a density of 500 and 600 cells/well, respectively. After adherence, the cells were treated with UA (0, 8, 16 or 32 μmol/L) for 48 h, then cultured for 15 days. The cells were then fixed with methanol and stained with a 5% Giemsa solution, and colonies (>50 cells) were counted under an inverted microscope. The reported results represent the average of 3 independent experiments performed over multiple days.

### Cell cycle analysis

GBC-SD and SGC-996 cells were seeded in 6-well plates at 1.5 × 10^5^ cells/well and treated with UA (0, 40, 50, 60 or 70 μmol/L) for 48 h. Then, the cells were collected, fixed with cold 70% ethanol and stored at −20°C. After thawing and centrifugation, the cells were washed with cold PBS, re-suspended and fixed in 70% ice-cold ethanol for 4 h at 4°C, then incubated at 37°C for 30 min with 10 mg/mL RNase. Finally, 1 mg/mL propidium iodide (Sigma-Aldrich) was used to stain the cells. DNA content analysis was performed using flow cytometry (San Diego, BD, USA). The percentage of cells present in the different cell cycle phases was determined using Cell Quest acquisition software (BD Biosciences).

### Flow cytometric analysis of cell apoptosis

Cell apoptosis levels were measured with an Annexin V-FITC apoptosis detection kit (BD Biosciences, USA) in accordance with the manufacturer’s instructions. In brief, GBC-SD and SGC-996 cells (10^5^ cells/2 mL/well) were seeded in 6-well plates and incubated with different concentration of UA (0, 40, 50, 60 or 70 μmol/L) for 36 h. The cells were then collected, resuspended in 100 μL of 1× binding buffer containing 2.5 μL of FITC-conjugated annexin-V and 1 μL of PI (100 μg/mL) and incubated for another 15 minutes in the dark. The results were then analyzed using flow cytometry (San Diego, BD, USA).

### Detection of variation in the mitochondrial membrane potential (ΔΨm)

ΔΨm was analyzed by flow cytometry using Rhodamine 123 staining. After treatment with UA (0, 40, 50, 60 or 70 μmol/L) for 48 h, the culture medium was removed, and cells were washed twice with PBS. The cells were then incubated in Rhodamine 123 staining solution (5 μg/mL) for 25 min at 37°C. The samples were analyzed using a flow cytometer (BD, Biosciences, USA).

### Western blotting analysis

GBC-SD and SGC-996 cells (1 × 10^7^) were seeded in a cell culture dish and treated with different concentrations of UA for 48 h. A BCA assay (Shanghai, Beyotime, China) was used to determine cell protein concentrations after harvesting the treated cells and extracting proteins using RIPA buffer according to the method described by Levites et al. [25]. For western blot analysis, equal quantities (50 μg of protein per lane) of total proteins mixed with bromophenol blue (0.01%) were added in each lane and separated by SDS-PAGE, then electrophoretically transferred onto PVDF membranes. The membrane was blocked in blocking buffer (5% non-fat dry milk) for 1 h at room temperature, then incubated with anti-cleaved caspase-3, anti-cleaved caspase-9, anti-PARP, anti-Bcl-2, anti-Bax, anti-cytochrome c, and anti-β-actin antibodies in blocking buffer at 4°C overnight. This was followed by an incubation with a goat anti-rabbit/anti-mouse secondary antibody conjugated with horseradish peroxidase (1:5000; Abcam). After each incubation period, the membranes were washed three times with TBS/T. The immunoreactive bands were visualized using a chemiluminescent HRP substrate (ECL; GE Healthcare), then scanned and quantified with a Gel Doc 2000 (BioRad, USA). The results are representative of 3 independent experiments.

### *In vivo* efficacy of UA

Six- to eight-week-old mail athymic nude mice were purchased from Shanghai SLAC Laboratory Animal Co., Ltd. (Shanghai, China). They were maintained in a pathogen-free environment. All experimental procedures were preformed strictly follow the international ethical guidelines and the National Institutes of Health Guide concerning the Care and Use of Laboratory Animals and were approved by the institutional guidelines of Shanghai Jiaotong University (Shanghai, China). To establish a tumor xenograft model, 2x10^6^ GBC-SD cells in log-phase growth were suspended in 0.1 ml serum-free culture medium and subcutaneously injected into the left axilla of all nude mice. After 24 hours, 45 mice were randomly divided into 3 groups (15 mice/group). Mice in the first group were treated with vehicle solvent (10% DMSO and 90% PBS) intraperitoneally. UA in a dose of 16 mg/kg and 32 mg/kg was intraperitoneally injected into the mice of other two groups everyday. On day 22, all mice were sacrificed, the tumor was harvested and weighed. 5 mice in each group were randomly chose to be shown in the figure.

### Statistical analysis

Each experimental value was expressed as the mean ± standard deviation (SD). All statistical analyses were performed using SPSS16.0 software. The differences between groups were assessed by Student’s t-test and were considered significant when the p value was less than 0.05. Statistical significance is denoted as follows: *p < 0.05, **p < 0.01, and ***p < 0.001. All data points represent the mean of triplicate data points.
